# Determination of the Influence of Mechanical Properties of Capsules and Seeds on the Susceptibility to Feeding of *Mononychus pubctumalbum* in Endangered Plant Species *Iris aphylla* L. and *Iris sibirica* L.

**DOI:** 10.3390/s21062209

**Published:** 2021-03-22

**Authors:** Magdalena Śmigała, Krystyna Winiarczyk, Agnieszka Dąbrowska, Marcin Domaciuk, Marek Gancarz

**Affiliations:** 1 Institute of Biological Sciences, Maria Curie-Skłodowska University, Akademicka 19, 20-033 Lublin, Poland; magdalena.smigala@gmail.com (M.Ś.); marcin.domaciuk@poczta.umcs.lublin.pl (M.D.); 2 Botanical Garden, Maria Curie-Skłodowska University, Sławinkowska 3, 20-810 Lublin, Poland; agnieszka.dabrowska@poczta.umcs.lublin.pl; 3 Institute of Agrophysics, Polish Academy of Sciences, Doświadczalna 4, 20-290 Lublin, Poland; 4 Faculty of Production and Power Engineering, University of Agriculture in Kraków, Balicka 116B, 30-149 Kraków, Poland

**Keywords:** *Mononychus punctumalbum*, *Iris aphylla*, *Iris sibirica*, capsule wall, seed, puncture test, precision agriculture, PCA analysis

## Abstract

The aim of the study was to determine the correlation between the mechanical resistance of iris seed capsules and seeds to *Mononychus punctumalbum* foraging. The principal component analysis (PCA) demonstrated that the first main component referred to the variety type in 68%, and the second main component described the stage of the ontogenetic development of the plant in 26%. As indicated by the values of each parameter measured, all the parameters were found to exert a strong impact on the variability of the analyzed system. The occurrence of weevil infestation was also strongly but negatively correlated with seed wall thickness and capsule wall thickness. There was a correlation of seed max load and seed mass with the occurrence of the weevil. The analysis of the mechanical resistance of iris seed capsules (in June 9.28 N and September 6.27 N for *I. sibirica* and in June 6.59 N and September 2.94 N for *I. aphylla*) and seeds (in June 15.97 N and September 344.90 N for *I. sibirica* and in June 16.60 N and September 174.46 N for *I. aphylla*) showed significant differences between the terms and species. The PCA analysis revealed that the first variable was correlated with the occurrence of weevil foraging.

## 1. Introduction

Technologies based on various types of sensors are now widely used in many fields of both industry and science. The most common types are optical sensors used to determine the content and concentration of gases or liquids [1,2], temperature sensors to measure the course of thermal processes [3,4], humidity sensors to determine the humidity level in studied environments [3,5], electrochemical sensors to detect volatile organic compounds in raw materials and food products [6,7], or force sensors to measure the mechanical properties of raw materials and products of plant origin [4,8,9,10]. Sensors are increasingly being used in precision agriculture, which has recently been developing very quickly, also with the use of information technologies and other techniques [11,12,13]. In most of the studies presented above, the results were subjected to statistical principal component analysis (PCA) in order to interpret the experimental results. In the study conducted by Gancarz et al. [3], the principal component analysis (PCA) method was used to correlate the results obtained from the control of the dough fermentation and bread baking process, thanks to which differences were distinguished not only in the processes but also in the subsequent stages of the processes. In turn, in the study carried out by Marek et al. [6], the origin of coffee was distinguished on the basis of PCA analysis of the results of measuring the aromas of roasted coffee. On the other hand, the PCA analysis of results of a study on aromas and mechanical properties of stored bread performed by Rusinek et al. [7] allowed correlating the parameters obtained with the onset of bread spoilage due to the formation of mold, which in the initial phase was not yet visible but was detected with the use of an electronic nose and a force sensor for examining the flesh of stored bread. The list of applications is open and there are growing numbers of newer applications of both sensors and statistical methods, the combination of which can provide an explanation and a completely new interpretation of the problem under study.

Several decades of research into interactions between plants and arthropods have revealed that these organisms are involved in the struggle for survival, which is based on sophisticated mechanisms of perception, signaling, and defensive activation on the plant side and on effective suppression of defense mechanisms and chemical and behavioral adaptations on the arthropod side [14,15,16,17].

Because research on endangered species has insufficient results from this type of experiments conducted using various types of sensors, an attempt was made to correlate selected mechanical properties and the morphology of seed capsules and seeds with the occurrence of weevil feeding. Therefore, the endangered species was chosen, which, however, is quite numerous in nature. As shown in *Index Kewensis*, there are over 250 identified iris species with different habitat requirements. They comprise hydrophytes, xerophytes, mesophytes, psammophytes, calciphytes, and calciphobes. There are approximately 30 iris species growing in natural habitats in Europe, with three species occurring in Poland: the yellow iris (*Iris pseudacorus* L.), the Siberian iris (*I. sibirica* L.), and the leafless iris (*I. aphylla* L.) [18]. The latter two species were analyzed in the present study. An important factor limiting the numbers of both analyzed species is the parasitism of *M. punctumalbum*, which feeds in the iris capsule. According to the current literature reports, the greatest threat to iris populations is posed by infestations of seed capsules by the weevil (*Mononychus punctumalbum*) [19,20,21]. However, observations conducted in 2017 confirmed these reports only in the case of *I. sibirica*. They demonstrated that the weevil pierced seed capsules, which were a source of nutrients during insect development [22]. In the case of *I. aphylla*, the weevil foraged only on flowers and did not damage the seeds [23].

In the period of ecosystem stability, the emergence of invasive species and sustainable control of insect pests in agriculture pose challenges for the growing human population, and application of new methods in research on the interaction of plants and pests can help to solve these problems [24,25].

The aim of the study was to determine the correlation between the morphological and mechanical traits of seed capsules and seeds of *I. aphylla* and *I. sibirica* (Iridaceae) involved in resistance to *M. pumctumalbum* foraging.

An innovative approach is the use of a force sensor to determine the mechanical properties of seed capsules and seeds and the correlation of the results with the presence of the weevil.

## 2. Materials and Methods

### 2.1. Plant Materials

**Figure 1:**
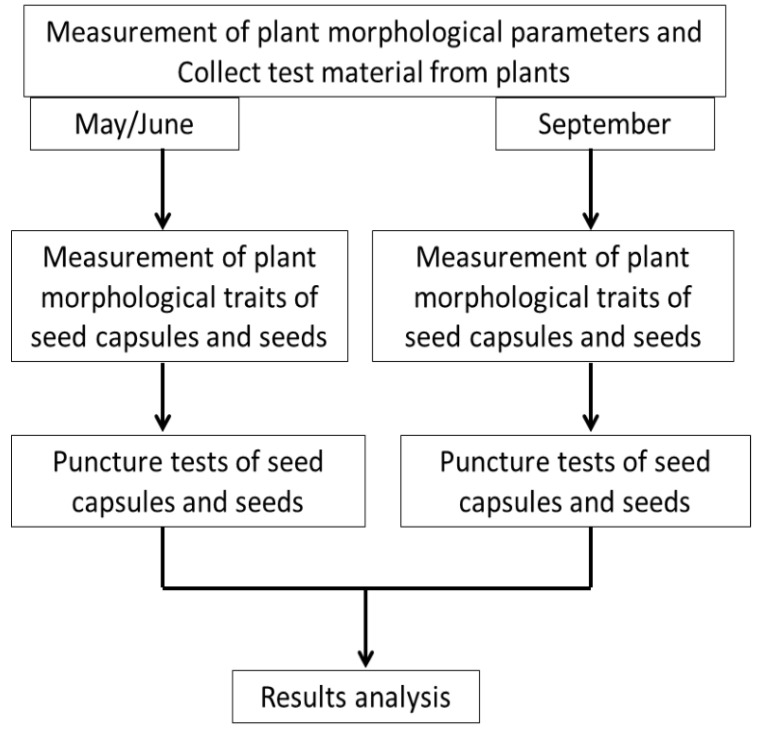
Diagram of the stages of the experiment.

The research material consisted of seed capsules and seeds of *I. aphylla* and *I. sibirica.* The plants were part of the collection of the UMCS Botanical Garden in Lublin, central-eastern Poland (51°14′37.2″ N, 22°32′25.3″ E; 197 m a.s.l.); therefore, no permission was needed to collect the material from the protected plants. The inventory numbers of the analysed *I. aphylla* and *I. sibirica* are as follows: Kazimierz Dolny 32/2007S, Tarnogóra 3190, Szczecyn near Gościeradów 4266P, Zawadówka near Chełm 3341E. Near the Garden, there is a herbarium is at the Maria Curie-Skłodowska University, but neither of the examined species of iris are recorded. Restructuring works on the herbarium are currently underway. The plants in the herbarium collection were marked over several decades [26,27,28]. The material was sampled in May/June (plant flowering and weevil infestations) and September (material harvesting). Figure 1 shows a diagram of the stages of the experiment.

### 2.2. Morphological Traits of Seed Capsules and Seeds

The morphological analyses of the colour and size of the seed capsules and seeds were performed on fresh material using a Nikon D500 camera and an Olympus SZ51 stereo microscope. Seeds intended for the observations in the SEM scanning electron microscope were prepared with the method proposed by Talbot and White [29]. The material was fixed in methanol for 10 min, rinsed in ethanol 2 × 30 min, dried in a CO_2_ atmosphere, sputter-coated with gold, and viewed in a scanning electron microscope LEO1430VP with a 15-kV accelerating voltage. Photographic documentation was made with the use of INCA-Mapping software (Billerica, MA, USA).

### 2.3. Puncture Tests

The seed capsules and seeds were subjected to puncture tests using the INSTRON 8872 fatigue test system (825 University Ave, Norwood, MA, USA) equipped with a measuring head with a maximum force of 250 N and 500 N in the case of seeds tested in September. A 2-mm diam. cylindrical penetration probe with a penetration rate of 10 mm/min was used in each test. Similar test parameters are used in many experiments to determine the relationship between the maximum puncture force and other material-specific parameters [30,31,32,33]. The capsule and the seed were pierced perpendicular to their surface. The tests were performed in ten replicates for each case and term of analysis. The following parameters were determined: maximum puncture force of the seed capsule (Capsule Max Load) and maximum puncture force of the seed (Seed Max Load).

### 2.4. Statistical Analysis

Correlation analysis and principal component analysis (PCA) were performed using Statistica software (version 12.0, StatSoft Inc., Tulsa, OK, USA) at a significance level of α = 0.05. PCA was used to determine the correlation of the assessed parameters with the iris varieties and term of analysis. The optimal number of the main components obtained in the PCA analysis was determined on the basis of Cattell’s criterion. The PCA data matrix had 8 columns and 40 rows. The input matrix was scaled automatically.

## 3. Results and Discussion

### 3.1. Characteristics of Seed Capsules and Seeds

The following parameters of the species were assessed in the experiment: Plant h [cm]—average plant height; seed mass per capsule [g]—average weight of seeds in the seed capsule; number of seeds per capsule—average number of seeds in the capsule; seed wall thickness [mm]—average thickness of the seed wall; seed mass [g]—average seed weight; capsule wall thickness [mm]—average thickness of the capsule wall; capsule max load (N)—average maximum force of seed capsule puncture; seed max load (N)—average maximum force of seed puncture.

The observations showed that *I. aphylla* was the first to start flowering, i.e., in the first half of May [34]. In turn, *I. sibirica* flowered in the second half of the month. The flowering shoots in *I. sibirica* were 120 cm high (Table 1).

They were substantially higher than the shoots of *I. aphylla*, which reached only approx. 35 cm (Table 1). Seed capsules were visible on average a month after flowering. They were composed of three chambers and differed in size; in *I. aphylla*, they were 4.3 ± 0.6 cm long, 2.7 ± 0.5 cm wide, and 1.7 ± 3 mm thick (Table 1). They were larger than the *I. sibirica* seed capsules, which were 2.6 ± 0.6 cm long, 1.4 ± 0.5 cm wide, and 0.6 ± 0.22 mm thick (Table 1). The surface of the *I. sibirica* seed capsule was evidently damaged by the weevil *M. punctumalbum*. The damage was visible in the interior of many seeds, which is a source of nutrient-rich food.

The macroscopic observations did not reveal any damage to the *I. aphylla* capsules; under the binocular, there were only single punctures, which turned out to be only superficial with no damage to the capsule wall. The seed capsules burst open at various time points, i.e., in July in *I. aphylla* and after the winter dormancy period in *I. sibirica*, which thus provides a perfect shelter for weevil generations. Iris seed capsules contained many seeds: 81 ± 12 in *I. aphylla* and 138 ± 19 in *I. Sibirica* (Table 1). The *I. aphylla* seeds did not fill the entire capsule but were located in its central part. The cross-section of the *I. sibirica* capsule showed that it was filled with seeds completely.

### 3.2. Puncture Tests Results

**Figure 2:**
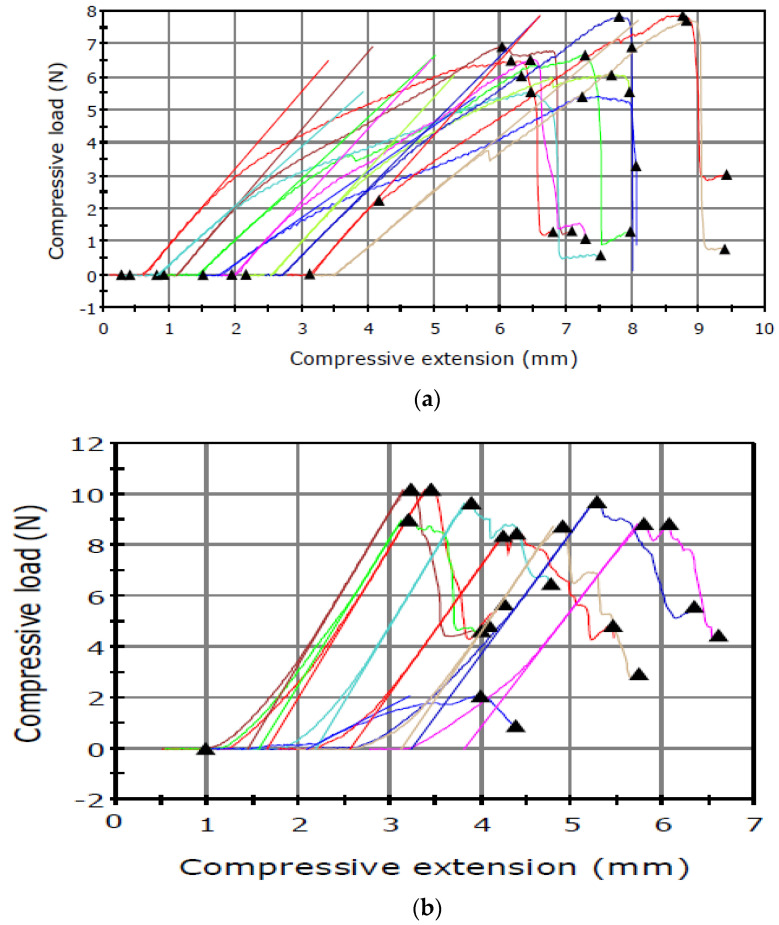

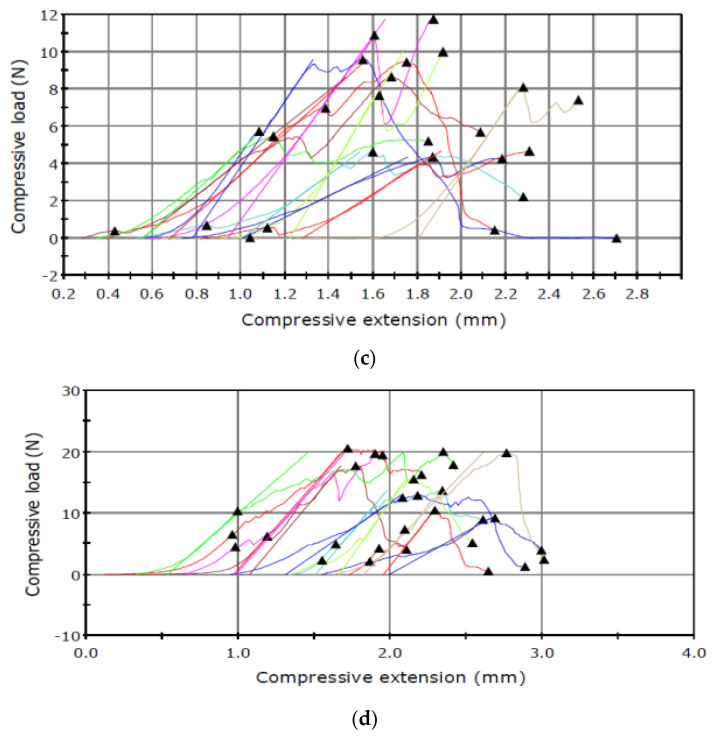
Puncture test carried out in June for the seed capsule of (**a**) *I. aphylla* and (**b**) *I. sibirica* and for the seeds of (**c**) *I. aphylla* and (**d**) *I. sibirica*.

**Figure 3:**
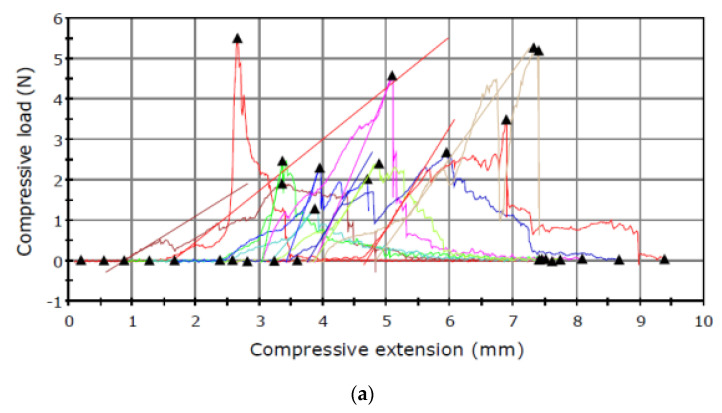

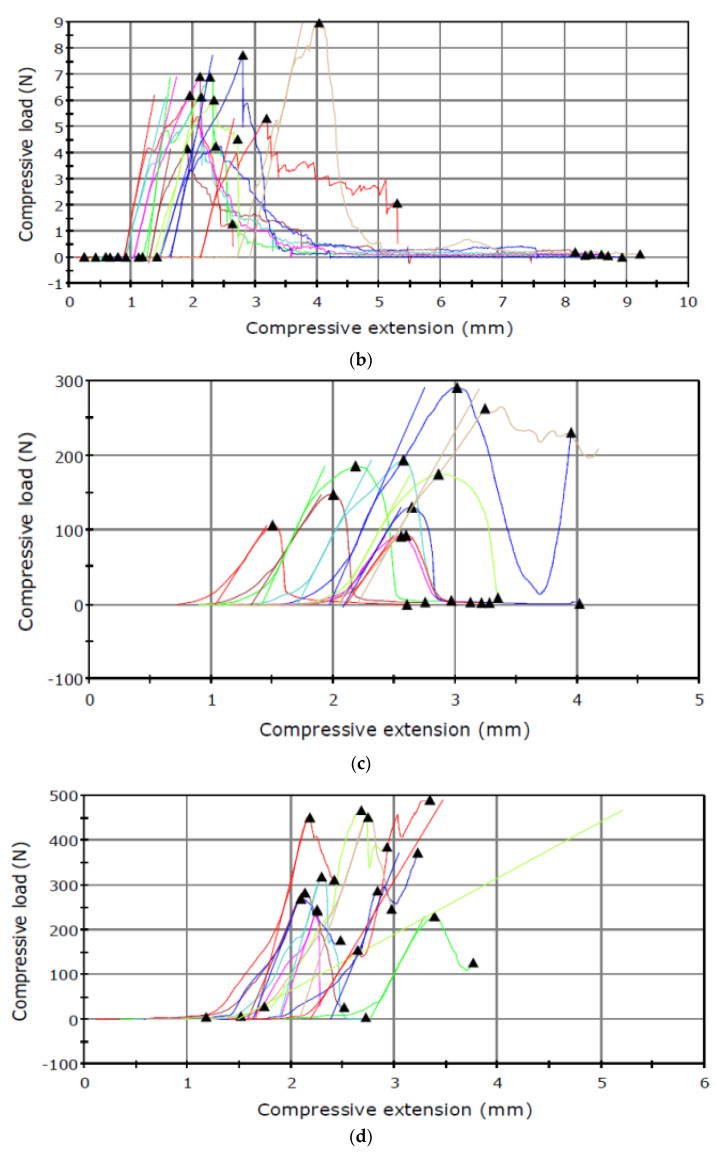
Puncture test carried out in September for the seed capsule of (**a**) *I. aphylla* and (**b**) *I. sibirica* and for the seeds of (**c**) *I. aphylla* and (**d**) *I. sibirica*.

The puncture tests revealed relationships between the maximum load and the type of the sample tested (Figure 2 and Figure 3).

The results presented in Table 1 shows higher capsule maximum load values obtained for the material analysed in June than in September. An inverse relationship was noted in the analysis of the seed maximum load, which had lower values in June and substantially higher results in September.

### 3.3. Principal Component Analysis of Characteristic Parameters

The PCA analysis separated the data into eight main components describing 100% of the variability, with the first two main components explaining as much as 95.12% of the variability of the entire system. Hence, the other components do not have such a large effect and can be disregarded in the further description of the results.

**Figure 4:**
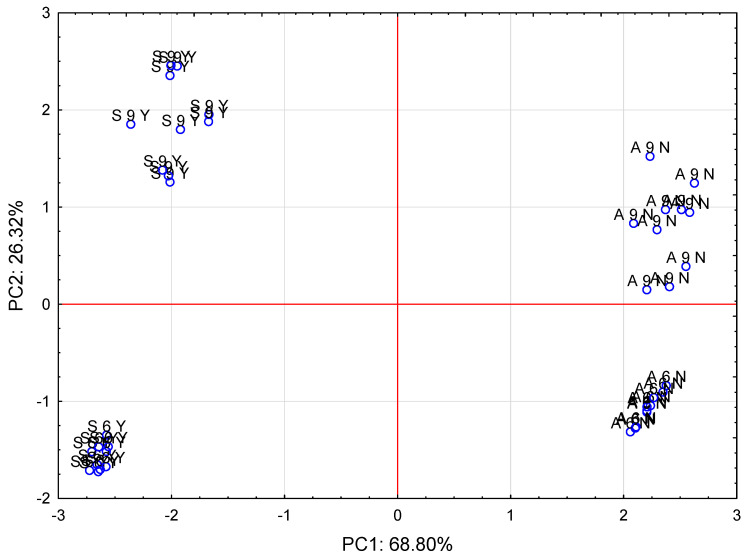
Plot of the PCA analysis results for two main components (PC1, PC2) obtained from seven parameters analysed in the investigated cases (A6N—*I. aphylla* collected in June, no foraging; A9N—*I. aphylla* collected in September, no foraging, S6Y—*I. sibirica* collected in June, foraging, S9Y—*I. sibirica* collected in September, foraging).

The analyzed cases differ from each other in terms of both the variety and the time of analysis/plant ontogenetic development, as shown in Figure 4.

**Figure 5:**
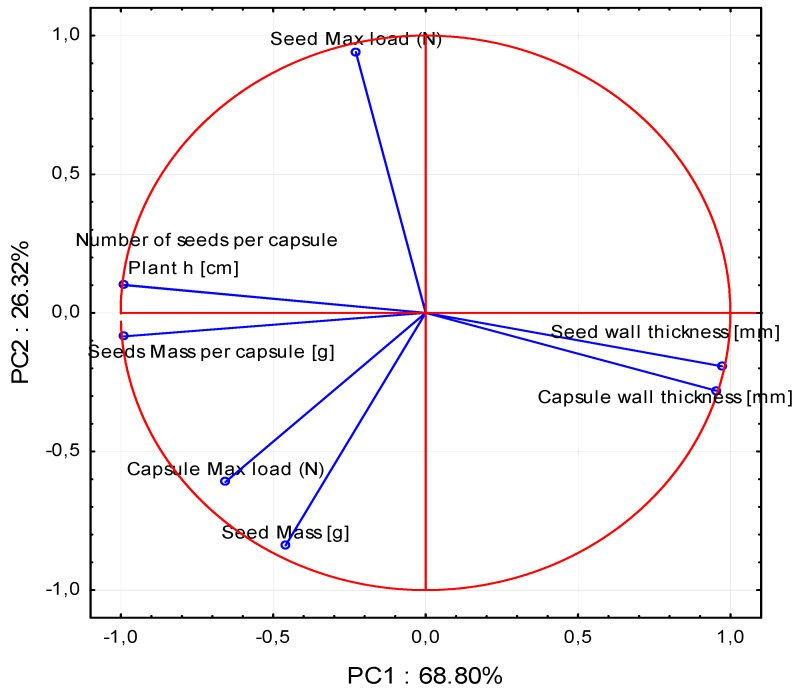
Graph of variables (Plant h [cm]. Seed mass per capsule [g]. Number of seeds per capsule. Seed wall thickness [mm]. Seed mass [g]. Capsule wall thickness [mm]. Capsule max load (N). Seed max load (N)). Position of load vectors relative to the first two PCA components.

This is evidenced by their distribution in the different quarters of the observations projected onto the first two components, which explain over 95% of the variability of the original data. Based on the PCA analysis, it is possible to analyze the original data in two dimensions with a very close approximation. It can be concluded that the first main component describes the species of the plant in over 68%, and the second main component refers to the age of the plant (term of analysis) in over 26%. The impact of the respective parameters is presented in Figure 5.

It is also possible to visualize the effect of the variables on the individual main components. Each of these variables is represented as a vector in Figure 5. The direction and length of this vector determines the extent to which each variable affects the individual main components. Such vectors are called loading vectors.

A strong positive correlation was observed between Plant h [cm], seed mass per capsule [g], and number of seeds per capsule. Similarly, there was a strong positive correlation between seed wall thickness [mm] and capsule wall thickness [mm]. There was no correlation of seed mass [g] and seed max load (N) with the following parameters: Plant h [cm], seed mass per capsule [g], number of seeds per capsule, seed wall thickness [mm], and capsule wall thickness [mm]. In turn, there was a strong negative correlation of Plant h [cm], seed mass per capsule [g], and number of seeds per capsule with seed wall thickness [mm] and capsule wall thickness [mm]. Additionally, there was a weak negative correlation of capsule max load (N) and seed mass [g] with such parameters as seed wall thickness [mm], capsule wall thickness [mm], and seed max load (N). A weak positive correlation was noted for capsule max load (N) and seed mass [g] with the following group of parameters: Plant h [cm], seed mass per capsule [g], and number of seeds per capsule. Based on the length of the loading vector, it can be concluded that all the parameters analysed exert a strong effect on the variability of the analysed system.

The proposed research, which combines the mechanical properties of seed capsules and seeds with the feeding of the scarlet weevil, is a completely new approach in the study of endangered species [15,16]. As indicated the PCA analysis, all examined parameters have a significant influence on the feeding or nonfeeding of the weevil. A very large influence of the seed max load on the second principal component (PC2) related to the growing season of the irises was observed. There was also a significant but smaller impact than that of the seed max load on the second principal component (PC2) of the capsule max load parameter related to the mechanical properties of the tested samples. The capsule max load also had a significant impact on the first principal component (PC1) (Figure 4 and Figure 5). To date, hardness tests have been performed mainly on vegetables or fruits containing large amounts of water and those with low water content, e.g., cereal seeds, legume plants, wood etc. [35,36,37,38,39,40,41,42]. Interestingly, this parameter has often been assessed using a conventional scale, as in the case of pomegranate (*Punica granatum* L.) [43].

Only the surface of the *I. sibirica* seed capsule was evidently damaged by the weevil *M. punctumalbum*. Such traits as the colour or size are commonly used in morphological analysis of floral elements, and the damage observed in *I. aphylla* and *I. sibirica* prompted deeper consideration of the harmful activity of *M. pumctumalbum* in different structures of iris flowers. In both analyzed species, an important factor limiting the numbers is the parasitism of *M. punctumalbum* feeding in the iris capsule [19,21,44,45]. Although *M. punctumalbum* feeds on the surface of *I. aphylla* capsules, it does not penetrate inside and cannot damage the seeds. Observations of copulation and frequent feeding of adult weevils on flowers falsely indicated *I. aphylla* as a host of *M. punctumalbum* in Central Europe. It fed and laid its eggs in *I. sibirica* capsules, where its larvae fed on the seeds. Although the seeds of *I. sibirica* are smaller than the seeds of *I. aphylla*, the total weight of healthy *I. sibirica* seeds per capsule is actually greater than that of *I. aphylla* seeds, and the seeds are a suitable food source for *M. punctumalbum* larvae. The capsule wall thickness and seed wall thickness in *I. aphylla* were greater than these parameters in *I. sibirica* (Table 1). The capsule wall thickness and seed wall thickness had the greatest influence on the first principal component defining *I. aphylla* (Figure 4 and Figure 5). The examination of the morphology of seed capsules conducted by Skuhrovec et al. [22] demonstrated that the weevil was not able to pierce the *I. aphylla* capsule due to its thickness, as the insect’s mouthparts are much shorter than the capsule wall thickness.

The attractive and fragrant *I. aphylla* flowers are the only source of nutrients and a mating site for the beetle, which is unable to damage the seeds. The observations showed that *I. aphylla* was the first to start flowering, i.e., in the first half of May [34]. In turn, *I. sibirica* flowered in the second half of the month. The flowering shoots in *I. sibirica* were 120 cm high (Table 1). The preliminary palpation examination of the seed capsules showed differences between the species, thus focusing the research on mechanical analysis. The *I. sibirica* seed capsules were harder than those of *I. aphylla*, which did not pose a problem to the weevil (Table 1, Figure 2 and Figure 3). As demonstrated by Woźniak [46] in a study of wheat seeds, the endosperm structure (mealy or vitreous) exerted a significant effect on the values of mechanical parameters. Seeds with vitreous endosperm exhibited higher mechanical strength than those with mealy endosperm. The harder capsule in *I. sibirica* opens much later than that of *I. aphylla*, which provides excellent conditions for the development of *M. punctumalbum* and constitutes a protective barrier for the juvenile stages of the insect. In the case of the tests performed, the thickness and hardness of the seed capsule determined whether the insect was able to penetrate this structure. In the case of *I. aphylla*, the hard wall was the main mechanical obstacle for the weevil. Moreover, the total weight of healthy seeds in *I. aphylla* was clearly lower than in *I. sibirica* and most probably insufficient for development of larvae. Similar results were reported by Yashchuk et al. [47], who determined the impact of the varietal traits of wheat grains on the degree of infestation by the grain weevil. The researchers proved that the difference in the infestation of grains of the analysed wheat varieties depended on the hardness of the grain. Dobrzański and Szot [35] conducted a study to assess the effect of different drying conditions on stress and deformation in the seed of three pea varieties. The modulus of elasticity was determined at a low deformation value, and comparable exponential regression parameters indicated similar mechanical properties of the seed test in the three varieties. *I. sibirica* had a much higher value of the seed max load parameter in the September term of research than *I. aphylla* (Table 1, Figure 3c,d). As suggested by other researchers, hard seeds are a source of mainly protein nutrients [30,31,48]. However, it is necessary to determine the endosperm composition using chemical tests, which will be part of the next stage of the research.

The interdisciplinary approach to the problem of the limited occurrence of the endangered species *I. aphylla* and *I. sibirica* in Poland will provide an unambiguous answer to the question of the causes of the decline in the iris population. In the future, it may contribute to conservation of these species or other plants in the Polish and global flora.

The present study shows issues for a wider audience—botanists, physicists, chemists, environmental protection, and agriculture. The methods used are not innovative, but we used them to show how an easily available and known method or a sensor can be used in completely new research on a specific material, e.g., protected plants. Environmental issues are extremely important, especially nowadays, and the use of even known methods to obtain new unambiguous and useful results is extremely important and should be presented to a wider group. In. addition, seemingly “unimportant” information about the studied iris species may be helpful for researchers who do not fully have an idea for solving their own problem. The results of the study may also be useful for scientists dealing with the problem of deterioration of the quality of agricultural materials caused by various insects, which leads to quality and economic losses of raw materials and products.

## 4. Conclusions

The study showed a relationship between the morphological and mechanical properties of seed capsules and seeds of *Iris aphylla* and *Iris sibirica* (Iridaceae) involved in resistance to *Mononychus pumctumalbum* foraging. Such traits as Plant height, number of seeds per capsule, and seed mass per capsule were strongly positively correlated with the occurrence of weevil foraging. A slight correlation was found between capsule max load and weevil foraging. The occurrence of weevil infestation was also strongly but negatively correlated with seed wall thickness and capsule wall thickness. There was a correlation of seed max load and seed mass with the occurrence of the weevil. Any new information on *M. punctumalbum* and its nutritional preferences on various species of irises in Central Europe can be used to verify literature data showing the important role of the one-year-old in the dramatic decline in the number of irises in natural habitats in Poland gardens, and other places. This is extremely important, as the iris population is threatened with extinction and any treatments that give the species a chance to survive should be investigated and applied. In the publication, the presented results explain the situation of the studied species. A potential reader can compare his research species in terms of similar occurrence, structure, or anatomy, which is extremely important for scientists involved in investigations of endangered or protected species. The results of the study may also be useful for scientists dealing with the problem of deterioration of the quality of agricultural materials caused by various insects, which leads to quality and economic losses of raw materials and products.

## Figures and Tables

**Table 1:** Morphological traits and mechanical properties of *I. aphylla* and *I. sibirica* seed capsules and seeds analyzed in June (6) and September (9).

Variety/Month	Capsule Max Load (N)	Seed Max Load (N)	Plant h [cm]	Capsule Wall Thickness [mm]	Seed Mass Per Capsule [g]	Seed Wall Thickness [mm]	Number of Seeds Per Capsule	Seed Mass [g]	Seed Length [mm]	Seed Width [mm]
*I. aphylla/*6	6.59	16.60	34.99	1.31	1.51	1.50	89.91	0.017	4.3	2.7
SD	0.92	29.72	0.08	0.01	0.01	0.01	0.83	0.0001	0.6	0.5
*I. aphylla/*9	2.94	174.46	35.01	1.1	1.35	1.22	90.11	0.015	4.0	2.5
SD	1.28	68.97	0.09	0.01	0.01	0.01	0.93	0.001	0.6	0.5
*I. sibirica/*6	9.28	15.97	120.01	0.45	3.0	0.10	148.80	0.02	2.6	1.4
SD	0.61	4.23	0.09	0.01	0.01	0.01	0.82	0.0001	0.6	0.5
*I. sibirica*/9	6.26	344.90	118.99	0.32	2.7	0.08	150.30	0.018	2.4	1.3
SD	1.48	95.81	0.09	0.01	0.01	0.01	0.94	0.001	0.6	0.5

Capsule max load (N)—average maximum force of seed capsule puncture. Seed max load (N)—average maximum force of seed puncture. Plant h [cm]—average plant height. Capsule wall thickness [mm]—average thickness of the seed capsule wall. Seed mass per capsule [g]—average weight of seeds in the seed capsule. Seed wall thickness [mm]—average thickness of the seed wall. Number of seeds per capsule—average number of seeds in the capsule. Seed mass [g]—average seed weight.

## Data Availability

The datasets used and/or analysed during the current study are available from the corresponding author on reasonable request.
